# Assessing Differential Transfusion Requirements for Children with Congenital Malformations vs. Pediatric Acute Abdomen Emergencies

**DOI:** 10.3390/diagnostics14192216

**Published:** 2024-10-04

**Authors:** Alin Ionescu, Alexandra Mihăilescu, Adela Chiriță-Emandi, Nitesh Munagala, Vlad Laurențiu David, Raluca Dumache, Dorel Săndesc, Ovidiu Bedreag, Roxana Folescu, Felix Bratosin, Paula Irina Barata, Dan-Mihai Cristescu, Mihai Alexandru Săndesc

**Affiliations:** 1 Center for Preventive Medicine, “Victor Babeș” University of Medicine and Pharmacy, 300041 Timișoara, Romania; ionescu.alin@umft.ro; 2 Doctoral School, “Victor Babeș” University of Medicine and Pharmacy, 300041 Timisoara, Romania; dancristescu93@gmail.com; 3 Centre of Genomic Medicine, Genetics Discipline, “Victor Babeș” University of Medicine and Pharmacy, 300041 Timisoara, Romania; alexandra.mihailescu@umft.ro (A.M.); adela.chirita@umft.ro (A.C.-E.); 4 Guntur Medical College Affiliated with Dr. NTR University of Health Sciences, Vijayawada 520008, India; munagalanitesh@gmail.com; 5 Department of Pediatric Surgery and Orthopedics, “Victor Babeș” University of Medicine and Pharmacy, 300041 Timisoara, Romania; david.vlad@umft.ro; 6 Center for Ethics in Human Genetic Identifications, “Victor Babeș” University of Medicine and Pharmacy, 300041 Timisoara, Romania; raluca.dumache@umft.ro; 7 Research Center CCATITM, “Victor Babeș” University of Medicine and Pharmacy, 300041 Timisoara, Romania; sandesc.dorel@umft.ro (D.S.); bedreag.ovidiu@umft.ro (O.B.); 8 Discipline of Family Medicine, “Victor Babeș” University of Medicine and Pharmacy, 300041 Timisoara, Romania; 9 Department of Infectious Disease, “Victor Babeș” University of Medicine and Pharmacy, 300041 Timisoara, Romania; felix.bratosin@umft.ro; 10 Center for Research and Innovation in Precision Medicine of Respiratory Diseases, “Victor Babeș” University of Medicine and Pharmacy, 300041 Timisoara, Romania; barata.paula@student.uvvg.ro; 11 Department of Physiology, Faculty of Medicine, “Vasile Goldis” Western University of Arad, 310025 Arad, Romania; 12 Research Centre of Timisoara Institute of Cardiovascular Diseases, “Victor Babes” University of Medicine and Pharmacy, 300041 Timisoara, Romania; 13 Research Center Professor Doctor Teodor Șora, “Victor Babeș” University of Medicine and Pharmacy, 300041 Timisoara, Romania; sandesc.mihai@umft.ro

**Keywords:** anemia, blood transfusion, congenital malformations, pediatric patients, children

## Abstract

Background and Objectives: This retrospective study aimed to evaluate the efficacy of preoperative blood transfusions in correcting anemia for pediatric patients with congenital malformations (CMs) versus those with acute abdomen (AA) conditions. The study hypothesized that the response to transfusions might vary significantly between these groups due to the differences in the underlying pathology and clinical status. Methods: The study included 107 pediatric patients admitted to Timisoara ‘Louis Turcanu’ Emergency Hospital for Children between January 2015 and May 2023, who required blood transfusions for preoperative anemia. Hemoglobin (HGB), hematocrit (HCT), and red blood cell counts (RBC) were assessed at admission, 48 h post-transfusion, and at discharge. Statistical analyses, including Student’s *t*-test, Pearson correlation, and chi-square tests, were utilized to compare outcomes between the groups. The study population was divided into 53 children with CM and 54 with AA. Results: Initial analyses showed that children with CM had statistically significantly higher baseline HGB (8.54 ± 1.00 g/dL vs. 7.87 ± 1.02 g/dL, *p* = 0.001) and HCT (26.07 ± 3.98% vs. 23.95 ± 2.90%, *p* = 0.002) compared to those with AA. Post-transfusion, children with CM exhibited a greater increase in HGB, with the highest increases noted in patients with central nervous system defects (mean increase of 3.67 g/dL, *p* = 0.038). In contrast, the increases in HGB for children with AA were less pronounced, with the highest being 2.03 g/dL in those with peritonitis (*p* = 0.078). Conclusions: No significant gender differences were noted in response to transfusion. Children with congenital malformations respond more effectively to preoperative blood transfusions compared to those with acute abdomen conditions. These findings suggest that differential transfusion strategies may be required based on the underlying medical condition to optimize the management of preoperative anemia in pediatric patients. Tailoring transfusion approaches according to specific patient needs and conditions could enhance clinical outcomes and resource utilization in pediatric surgical settings.

## 1. Introduction

Anemia is a significant and modifiable risk factor associated with elevated perioperative morbidity and mortality. It is estimated that a quarter of the world’s population is affected by anemia, with around 40% of children under five being diagnosed with anemia in 2023 [1,2].

It is essential to identify and, if possible, promptly correct the etiology of anemia, as it varies significantly among different age groups. In neonates, anemia is often due to hemorrhage and hemolysis (e.g., isoimmune hemolysis or glucose-6-phosphate dehydrogenase deficiency), rather than decreased production [3]. Additionally, congenital anomalies and conditions associated with blood loss, such as surgical procedures, can contribute to anemia in this age group. For example, a newborn who undergoes surgery is expected to develop anemia subsequently due to perioperative blood loss. In the pediatric population beyond the neonatal period, common causes of anemia include iron deficiency, infections, hemoglobinopathies, and other chronic conditions [1]. Iron deficiency remains one of the most critical causes of anemia in children worldwide. Gedfie et al. [4], in a systematic review, reported a global prevalence of iron-deficiency anemia of 16.42%, while the prevalence of iron deficiency reached 17.95% in children under 5 years of age. Moreover, they highlighted considerable variability among different geographic regions. Similar findings were reported by Nazari et al. [5], who found prevalences of 18.2% for iron-deficiency anemia and 27.7% for iron deficiency in Iranian children under 6 years of age. Certain causes of anemia, such as sickle cell disease and leishmaniasis, are significantly more prevalent in specific geographic areas. Therefore, geographic and age-related factors should be carefully considered, and a thorough history should be obtained [6,7].].

Regardless of the etiology, when a blood transfusion is necessary, blood requirements should be thoroughly calculated to avoid hypervolemia and other transfusion reactions. Thus, age, clinical status, and comorbidities, as well as weight and increment in hemoglobin, should all be considered before making clinical decisions [8,9].

Transfusion-associated circulatory overload (TACO) is a serious transfusion complication associated with increased morbidity and mortality [10]. It is characterized by pulmonary edema and congestive heart failure due to increased hydrostatic blood pressure from volume overload following blood transfusion [11]. However, overload and pulmonary edema are not common complications in children when transfused with the correct volumes at appropriate rates. The data regarding the prevalence of TACO in pediatric patients are inconsistent, partly due to varying definitions and patient populations studied. For instance, De Cloedt L et al. [12] reported incidence rates ranging from 1.5% to 76%, depending on the criteria used to define TACO and whether a 10% or 20% threshold increase in blood volume was considered. The wide range reflects differences in patient groups, including those with and without comorbidities that predispose them to circulatory overload. In children without comorbidities, the incidence of transfusion complications, including TACO, is significantly lower when transfusions are administered with careful attention to volume and rate. Therefore, adherence to appropriate transfusion protocols is essential to minimize the risk of TACO and other transfusion-related complications in pediatric patients.

The efficient treatment of anemia is crucial in patients scheduled for surgery since the condition has been associated with an increased risk of postoperative complications, longer hospitalization, and a higher mortality rate. In a study published by Faraoni et al. [13], preoperative anemia was associated with higher in-hospital mortality (OR 2.17) in children undergoing noncardiac surgery. Likewise, Scott et al. [14], in a meta-analysis of children aged between 28 days and 12 years, reported a 24% decrease in death risk for each 1g/dL increase in hemoglobin.

Depending on age categories, anemia can arise from various origins, such as congenital anomalies, conditions that predispose individuals to blood loss, surgical interventions, and chronic disease [15,16,17,18]. For instance, newborns undergoing surgical procedures often face a high risk of developing anemia due to intraoperative or postoperative blood loss. Moreover, the mechanisms responsible for the complications linked to anemia are related to the pathophysiological response to hypoxia. To illustrate, in the case of cerebrovascular events, the hyperkinetic state associated with anemia determines the hyperexpression of different adhesion molecules in endothelial cells, consequently increasing the risk of thrombi [19]. In addition, impaired oxygen delivery to organs reduces the tolerance to ischemia and hemorrhage. In bacterial infections, many pathways seem to act synergically (e.g., enhanced erythropoietic drive, hemolysis, immune dysfunction) [20].

The aim of the current study was to evaluate the differences in the efficacy of preoperative blood transfusions in correcting anemia between children with congenital malformations (CMs) and those with acute abdomen (AA). We hypothesized that children with CMs—chronic conditions allowing for planned transfusions—would exhibit better correction of anemia compared to children with AA, where the acute nature of the condition limits the time for preoperative optimization. By comparing these two groups, we aimed to determine whether the ability to plan transfusions impacts their effectiveness, despite the predictability suggested by the chronic versus acute distinction.

## 2. Materials and Methods

### 2.1. Participant Selection

The current study followed the PICO statement (Population, Intervention, Comparison, Outcome). Population: Pediatric patients admitted to the Timisoara ‘Louis Turcanu’ Emergency Hospital for Children requiring preoperative blood transfusions to correct anemia. Intervention: Blood transfusion to manage preoperative anemia in children with congenital malformations (CM). Comparison: Pediatric patients with acute abdomen (AA) conditions requiring similar preoperative blood transfusions. Outcome: Efficacy of blood transfusions in increasing hemoglobin levels, evaluated through hemoglobin (HGB), hematocrit (HCT), and red blood cell counts (RBC) assessed at admission, 48 h post-transfusion, and at discharge.

This retrospective study included 107 surgical pediatric patients admitted to Timisoara ‘Louis Turcanu’ Emergency Hospital for Children, who received preoperative blood transfusion between January 2015 and May 2023.

Inclusion criteria were represented by the requirement of blood transfusion for preoperative anemia correction.

Exclusion criteria were represented by the presence of associated malignancies and primary hematological disorders. Patients that had both diverticulitis and congenital diverticulum were also excluded from the current study.

We evaluated three consecutive hemograms for each participant: at admission (baseline), at 48 h’ post-transfusion, and upon discharge from the hospital.

Anemia was defined by the hemoglobin values, according to the definition of the World Health Organization (WHO): 12–59 months of age lower than 11.0 g/dL, 5–11 years of age <11.5 g/dL, 12–14 years <12.0 g/dL, female 15–17 years of age <12.0 g/dL, and male 15–17 years of age <13.0 g/dL. 

The study was approved by the Ethics Committee of the “Victor Babes” University of Medicine and Pharmacy Timișoara (68/01.10.2018 rev 2024), Romania, and conducted in accordance with the Declaration of Helsinki.

### 2.2. Laboratory Variables

A Sysmex XN-550 Hematology Analyzer (Kobe, Hyogo, Japan) was used for in vitro diagnostic use in determining whole blood diagnostic parameters. The method used fluorescence flow cytometry, flow cytometry, DC impedance method with hydrodynamic focusing, and cyanide-free SLS method. Parameters analyzed included: white blood cells (WBCs), red blood cells (RBCs), hemoglobin (HGB), hematocrit (HCT), mean corpuscular volume (MCV), mean corpuscular hemoglobin (MCH), mean corpuscular hemoglobin concentration (MCHC), platelet count (PLT), red cell distribution width (RDW), red cell distribution width–standard deviation (RDW-SD), red cell distribution width–coefficient of variation (RDW-CV), platelet distribution width (PDW), mean platelet volume (MPV), platelet–large cell ratio (P-LCR), platelets (PCTs), neutrophils (NEUTs), lymphocytes (LYMPHs), monocytes (MONOs), eosinophils (EOs), basophils (BASOs), NEUT%, LYMPH%, MONO%, EO%, BASO%, and immature granulocyte (IG). However, our analysis focused on RBC, HGB, and HCT, as markers of anemia.

### 2.3. Transfusions

Blood transfusions were initiated based on the WHO guidelines for hemoglobin concentrations that indicate severe anemia, with a general recommendation of 7 g/dL, which necessitates immediate correction to mitigate the risk of hypoxia and other complications. In cases of CM, transfusions were planned based on the anticipated blood loss during upcoming surgeries, considering the patient’s overall health and ability to tolerate anemia. Conversely, for AA, transfusions were often emergent, driven by acute blood losses and the need to stabilize the patient’s condition rapidly. Consistent with contemporary pediatric care standards, the protocol aimed to minimize blood transfusions. This was achieved by employing restrictive transfusion strategies, where transfusions were only given when necessary and in the smallest effective volumes to reduce risks of transfusion-related complications. In the initial transfusion, patients were closely monitored through serial hemograms measured at admission, 48 h post-transfusion, and at discharge. Adjustments to the transfusion plan were made based on these ongoing assessments to ensure optimal patient outcomes without excessive blood administration.

### 2.4. Statistical Analysis

Data analysis was performed using the statistical software IBM SPSS Statistics 25 (SPSS Inc. Chicago, IL, USA). The Kolmogorov–Smirnov test was used to determine the normality of data. The Student’s *t*-test was used to compare continuous variables between the two groups, and Pearson correlation coefficient was used to assess associations between certain variables. Chi-square test was used to assess the differences in sex distribution between CM and AA groups. Age was accounted for by including it as a covariate in multivariate regression models evaluating the efficacy of blood transfusions. Additionally, we conducted subgroup analyses within specific age brackets to further assess the impact of age on transfusion outcomes. By adjusting for age, we aimed to ensure that any observed differences in transfusion efficacy between the CM and AA groups were attributable to the clinical conditions rather than age disparities between the groups. Continuous variables were compared using the Student’s *t*-test for normally distributed data or the Mann–Whitney U test for non-normally distributed data, while categorical variables were compared using the chi-square test. A *p*-value of <0.05 was considered statistically significant.

## 3. Results

### Demographics

The patients were aged between 1 day to 17 years. The cohort was divided into two subgroups according to their diagnosis: 53 participants with congenital malformations (diaphragmic hernia, esophageal atresia, gastroschisis, Hirschsprung disease, spina bifida, and congenital hydronephrosis) and 54 participants with anemia that were diagnosed with acute abdomen (acute appendicitis, peritonitis, diverticulitis, intestinal obstruction, and trauma-related blood loss). A more detailed overview of the patients is presented in Table 1.

The mean age of the subjects included in this study was 29.86 ± 56.67 months, while gender distribution showed predominantly males 70/107 (65%). The mean values of age, hospitalization period, and sex distribution are shown in Table 2. While males are predominant in both groups, their weighting is significantly higher in the Congenital Malformation group (71.70%) in comparison to the Acute Abdomen group (59.26%). Additionally, there is a statistically significant difference regarding age (*p* < 0.001) with patients from the CM group being younger than those from the acute abdomen group.

**Figure 1:**
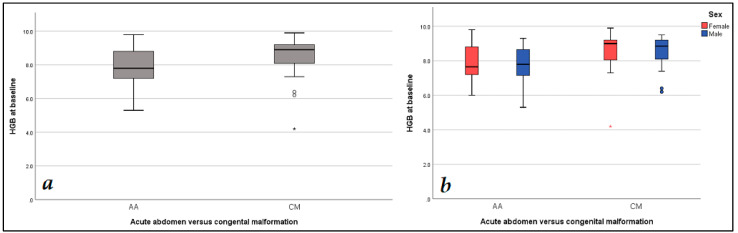
Hemoglobin level values: (**a**) baseline hemoglobin level AA vs. CM groups; (**b**) baseline hemoglobin level comparison according to gender and diagnosis.

**Figure 2:**
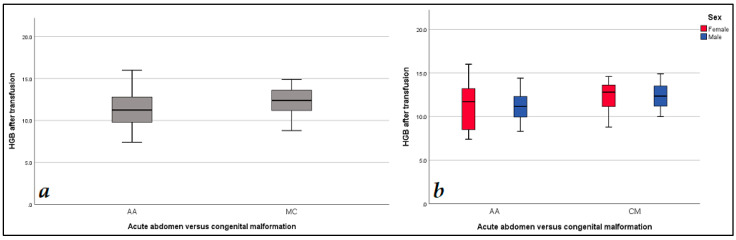
Hemoglobin level values: (**a**) hemoglobin at the 48 h follow-up AA vs. CM groups; (**b**) hemoglobin at the 48 h follow-up level comparison according to gender and diagnosis.

**Figure 3:**
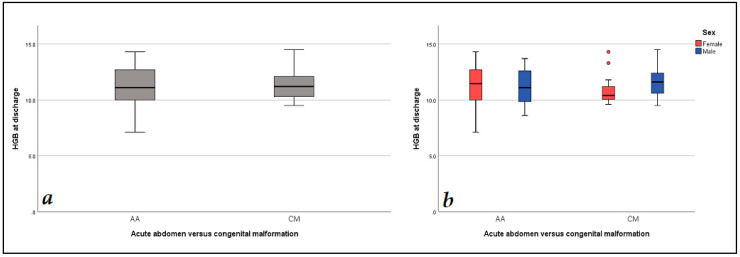
Hemoglobin levels values: (**a**) hemoglobin level at discharge level comparison between AA and CM groups; (**b**) hemoglobin level at discharge regarding gender and diagnosis.

Data analysis revealed statistically significant lower values of the baseline level of hemoglobin (*p* = 0.001) and hematocrit (*p* = 0.002) in the AA versus the CM group. In a likewise manner, differences in the aforementioned parameters were observed at the 48 h follow-up (*p* = 0.001 and *p* = 0.005, accordingly). These findings are presented in Table 3. The variation in hemoglobin levels at admission, at the 48 h follow-up and at discharge with regard to diagnosis and gender is presented in Figure 1, Figure 2 and Figure 3. When the cohort was divided by gender into two groups, males (N = 70) and females (N = 37), the data analysis did not find any statistically significant differences regarding both demographic data and blood parameters (Table 2 and Table 3). At discharge, 57% of all subjects had anemia, with no differences between AA and CM groups.

The data analyzed from Table 4 indicated statistically significant differences in blood parameters between the CM and AA groups, stratified by age. For each age category, the CM group consistently demonstrated higher levels of RBC, HGB, and HCT compared to the AA group. Notably, in the youngest cohort (0–12 months), CM patients exhibited a mean HGB level of 8.67 g/dL, significantly higher than the 7.78 g/dL observed in AA patients, with a *p*-value of 0.001, suggesting a robust difference in the initial hemoglobin status between these groups. The statistical tests employed, including *t*-tests for comparing means and chi-square tests for comparing proportions, consistently showed these differences across all age categories. Similarly, the anemia rates at discharge were lower in the CM group across all ages, with *p*-values consistently below 0.05, indicating a statistically significant better outcome in anemia management for the CM group compared to the AA group.

The data presented in Table 5 show a negative correlation between the baseline level of hemoglobin and age. The strength of the association varies between groups: weak in the AA group (r = −0.378, *p* = 0.005), strong in the CM group (r = −0.611), and moderate at the cohort level (r = −0.469). In addition, a positive correlation between the level of hemoglobin before and after blood transfusion was found in the CM group (r = 0.423, *p* = 0.002) and at the cohort level (r = 0.392).

In the multivariate analysis of factors affecting hemoglobin correction post-transfusion in pediatric patients, significant differences were observed between children with congenital malformations (CMs) and those with acute abdomen (AA) conditions. Patients in the AA group demonstrated a statistically significant decrease in hemoglobin correction compared to the CM group, with a coefficient of −0.87 (*p* = 0.004). This finding suggests that children with acute abdomen may have less effective anemia correction through transfusion, which could be due to factors such as ongoing blood loss or a more severe initial state of anemia. Additionally, the analysis indicated that age negatively influenced hemoglobin correction, with each additional month of age reducing hemoglobin levels by 0.02 g/dL (*p* = 0.037), highlighting a lesser efficacy of transfusion in older children within the studied cohort.

The study also explored the roles of gender and other variables in transfusion outcomes. Females tended to have higher increases in hemoglobin levels post-transfusion compared to males, although this difference did not reach statistical significance (coefficient = 0.54, *p* = 0.112). Conversely, higher baseline hemoglobin levels significantly predicted better post-transfusion outcomes, with an increase of 0.49 g/dL for each unit increase in initial hemoglobin (*p* < 0.001). Additionally, a longer hospitalization was associated with better hemoglobin recovery, with each additional day linked to an increase of 0.07 g/dL in hemoglobin levels (*p* = 0.028). These results underscore the complexity of factors influencing the efficacy of blood transfusions in pediatric surgical patients, suggesting that individual patient characteristics and clinical settings play critical roles in determining transfusion requirements and outcomes (Table 6).

In the subgroup analysis of hemoglobin correction post-transfusion, children diagnosed with congenital malformations (CMs) generally exhibited higher mean increases in hemoglobin levels compared to those with acute abdomen (AA) emergencies. Specifically, central nervous system defects within the CM category showed the most significant improvement in hemoglobin levels, with a mean increase of 3.67 g/dL (*p* = 0.038). This statistically significant finding indicates a robust response to transfusion in this subgroup. In contrast, gastrointestinal tract malformations and diaphragmatic hernias also showed increases, 3.24 g/dL and 2.98 g/dL respectively, but the changes were not statistically significant (*p* = 0.052 and *p* = 0.112, respectively), suggesting variable efficacy of transfusions within different types of congenital malformations.

On the other hand, children in the AA group had lower mean increases in hemoglobin levels following transfusion, with none reaching statistical significance. The highest increase was observed in children with peritonitis, who showed a mean increase of 2.03 g/dL (*p* = 0.078), followed by intestinal obstruction and appendicitis with increases of 1.89 g/dL (*p* = 0.093) and 1.76 g/dL (*p* = 0.101), respectively. These results suggest that children with AA conditions may have a less effective response to transfusions, possibly due to ongoing blood loss or the acute nature of their conditions. This differential response underscores the need for tailored transfusion strategies based on the specific underlying condition and severity of anemia at presentation, as presented in Table 7.

## 4. Discussion

Our study evaluated the efficacy of preoperative blood transfusions in correcting anemia among pediatric patients with congenital malformations (CMs) and those with acute abdomen (AA), providing new insights into how the chronic nature of CMs and the acute presentation of AA impact transfusion outcomes. In CM patients, scheduled transfusions led to a significant increase in hemoglobin levels 48 h post-transfusion, with a mean increase of 3.81 g/dL (from 8.54 ± 1.00 g/dL to 12.35 ± 1.49 g/dL, *p* < 0.001). Conversely, in the AA group, although the urgency limits preoperative optimization, transfusions still resulted in a meaningful hemoglobin increase of 3.33 g/dL (from 7.87 ± 1.02 g/dL to 11.20 ± 2.05 g/dL, *p* < 0.001), albeit to a lesser extent than in CM patients. The magnitude of hemoglobin correction was significantly greater in the CM group compared to the AA group (coefficient = −0.87, *p* = 0.004), highlighting challenges in managing anemia in acute settings. Within-group analyses identified that in CM patients, factors like baseline anemia severity and comorbidities affected transfusion outcomes, while in AA patients, factors such as the extent of acute blood loss and hemodynamic instability played significant roles. These findings suggest that optimizing transfusion timing in CM patients enhances anemia-correction efficacy, whereas in AA patients, the acute nature of their condition may limit the effectiveness of transfusions despite timely intervention.

Anemia is a relatively common finding in patients undergoing surgery, detected during the initial assessment or postoperative care. Preoperative anemia can be found in approximately 30–40% of the patients undergoing major surgery, while the prevalence of postoperative anemia reaches up to 90% [21]. Perioperative anemia has been assessed as an independent risk factor for perioperative morbidity and mortality by numerous studies. Anemia has been associated with poor clinical outcomes, prolonged hospitalization, increased need for ICU care, higher mortality, and an increased rate of complications [22,23,24]. Careful surveillance and timely identification of at-risk patients enable adequate preoperative treatment, such as iron supplementation (for non-critical patients qualifying for procedures) or blood transfusions (requiring emergency surgery). Nonetheless, the appropriate treatment will be chosen considering the etiology, clinical status, age, weight, particularities, and bleeding risks associated with the surgical procedure required.

The sex distribution in our study was uneven, with a significantly higher number of male subjects in both the AA and CM groups. However, the proportion of males was significantly bigger in the CM group (70.70%) compared to the AA group (59.26%) (chi-square test *p* < 0.001). Numerous studies presented in the literature highlighted the role gender plays as a risk factor for congenital malformations. For instance, Tennant et al. [25], in a study on 12.795 cases of congenital anomalies, reported that males accounted for 54.9% of cases. Moreover, when compared to female fetuses, pregnancies with male fetuses were associated with a higher risk of congenital anomalies (RR, male vs. female = 1.15). Sokal et al. [26], in a population-based study that included 794.169 children (born between 1990 and 2009), reported that the overall risk for congenital anomalies was 26% higher in male versus female subjects (PR [M:F] 1.26). Likewise, Shaw et al. [27] reported that the prevalence of birth defects was 22% higher among males. Lary et al. [28] examined the records for 1968 through 1995 from the Metropolitan Atlanta Congenital Defects Program in order to assess the sex-specific prevalence of different congenital disabilities. Their findings suggest that females have a higher prevalence of nervous and endocrine system anomalies, while males are at a higher risk for all the other birth defects. For example, gastrointestinal tract defects and urinary tract anomalies were 55% and 67%, respectively, more prevalent among male subjects.

Our results indicated a negative correlation between age and hemoglobin baseline levels in both groups, especially in the CM group, showing that younger age is correlated with higher blood count values. Several reasons could explain this phenomenon. Firstly, the differences in the etiology of anemia might provide a plausible explanation since the acute abdomen can be associated with a significantly higher or faster blood loss. Secondly, there is a physiological difference in red blood cell count and erythrocyte morphology in neonates compared to adults. For instance, the volume of red blood cells is 21% larger, and the diameter is 13% bigger in neonates compared to adults [29]. Younger age (neonatal presentation) was more prevalent in the congenital malformation group, as they were observed immediately after birth. To illustrate, Bower et al. [30] analyzed the Western Australian Birth Defects Registry data. They reported that in 18.7% of cases, the birth defects were diagnosed prenatally, while a further 47.8% were diagnosed by the age of 1 month. Zhu et al. [31] reported a diagnosis rate at birth of 29.7%. Hemoglobin levels at baseline positively correlated with the values after transfusion, further showing that milder anemia is easier to correct with blood transfusions.

There were statistically significant differences regarding the prevalence of anemia at discharge between the AA and CM groups or between male and female groups. This is probably because all children were treated following similar guidelines and treatment protocols and benefited from identical healthcare standards.

The novelty of our study lies in the detailed examination of transfusion efficacy within specific pediatric surgical populations and the identification of factors that influence outcomes in each group. While it is generally understood that scheduled transfusions are preferable when time permits, our study quantifies this advantage and underscores the importance of individualized transfusion strategies. Our findings suggest that in CM patients, proactive anemia management should be a priority to optimize surgical outcomes. In AA patients, despite the limitations imposed by the acute nature of their condition, efforts should be made to correct anemia as effectively as possible, perhaps by exploring rapid-acting interventions or protocols tailored to emergency settings.

The limitations of the current study consist in the relatively small number of subjects (107), as well as the lack of data regarding additional parameters like serum iron level, ferritin, serum folate and cobalamin levels, etc., that could prove useful in identifying additional predictors for blood-transfusion requirements. One should also take into account the possibility that patients with congenital malformations might experience difficulties in absorbing nutrients, which can result in malnutrition and anemia. Moreover, ongoing blood loss or a more severe initial state of anemia may be influenced by various confounding factors, such as the differences in blood transfusion principles among surgeons. Future studies that use larger cohorts, with either pediatric or adult participants, from more areas of the world are welcomed additions to this theme, mainly if they include further knowledge of patient blood-management strategies that can reduce the need for transfusion in anemia patients undergoing surgeries.

## 5. Conclusions

In this study, the children suffering from acute abdomen presented more severe anemia before the transfusion, compared to those with congenital malformations. In addition, the anemia was more difficult to correct in the acute abdomen group, suggesting that careful surveillance is needed for this group. No statistically significant differences regarding blood parameters were observed between genders, suggesting that gender does not influence the efficacy of blood transfusion at this age.

## Figures and Tables

**Table 1:** Patients’ diagnostics identified in the study cohort.

Group	Gender	Diagnosis	Count	Total
CM	F	Gastrointestinal tract malformation	12	15
		Central nervous system defect	2	
		Cleft palate	1	
	M	Gastrointestinal tract malformation	27	38
		Urinary tract malformation	5	
		Diaphragmatic hernia	2	
		Cleft palate	1	
		Central nervous system defect	2	
		Pulmonary sequestration	1	
AA	F	Gastrointestinal tract hemorrhage	2	22
		Intraperitoneal bleeding	2	
		Appendicitis	5	
		Peritonitis	5	
		Intestinal obstruction	7	
		Necrotizing enterocolitis	1	
	M	Gastrointestinal tract hemorrhage	2	32
		Intraperitoneal bleeding	10	
		Appendicitis	3	
		Peritonitis	6	
		Intestinal obstruction	9	
		Necrotizing enterocolitis	2	

**Table 2:** Cohort demographics (gender distribution, age, hospitalization period).

Variables	All N = 107	Female N = 37	Male N = 70	*p*-Value	CM N = 53	AA N = 54	*p*-Value
Gender F/M N, (%)/N, (%)	37 (34.58%)/70 (65.42%)	37	70	-	15 (28.30%)/38 (71.70%)	22 (40.74%)/32 (59.26%)	<0.001 *
Age in months	29.86 ± 56.67	41.06 ± 66.35	23.93 ± 50.32	0.175 **	16.94 ± 23.94	52.34 ± 69.42	<0.001 **
Hospitalization days (mean, ± SD)	17.02 ± 12.94	15.11 ± 7.47	18.03 ± 15.00	0.182 **	15.08 ± 7.99	18.93 ± 16.27	0.123 **

* Chi-Square Test; ** *t* test.

**Table 3:** Blood parameter comparison between groups.

Variables	All N = 107	Female N = 37	Male N = 70	*p*-Value	Congenital Malformation N = 53	Acute Abdomen N = 54	*p*-Value
Baseline evaluation							
RBC (×10^6^/mm^3^)	2.89 ± 0.42	2.82 ± 0.49	2.92 ± 0.36	0.307 **	2.89 ± 0.42	2.88 ± 0.40	0.927 **
HGB (g/dL)	8.21 ±1.07	8.11 ± 1.25	8.25 ± 0.95	0.560 **	8.54 ± 1.00	7.87 ± 1.02	0.001 **
HCT (%)	25.01 ±3.62	24.66 ± 4.45	25.19 ± 3.11	0.521 **	26.07 ± 3.98	23.95 ± 2.90	0.002 **
Evaluation at 48 h following the transfusion							
RBC (×10^6^/mm^3^)	4.05 ±0.66	3.95 ± 0.77	4.09 ± 0.59	0.340 **	4.09 ± 0.56	4.00 ± 0.74	0.451 **
HGB (g/dL)	11.77 ± 1.88	11.62 ± 2.27	11.85 ± 1.64	0.596 **	12.35 ± 1.49	11.20 ± 2.05	0.001 **
HCT (%)	34.26 ± 4.96	33.74 ± 6.14	34.52 ± 4.23	0.491 **	35.60 ± 4.13	32.94 ± 5.37	0.005 **
Evaluation at discharge							
RBC (×10^6^/mm^3^)	3.99 ± 0.69	3.87 ± 0.73	4.05 ± 0.66	0.204 **	3.90 ± 0.59	4.07 ± 0.77	0.204 **
HGB (g/dL)	11.30 ± 1.55	11.04 ± 1.78	11.43 ± 1.40	0.256 **	11.43 ± 1.33	11.16 ± 1.72	0.359 **
HCT (%)	33.49 ± 4.32	32.86 ± 5.10	33.81 ± 3.84	0.325 **	33.47 ± 3.89	33.49 ± 4.73	0.984 **
Anemia at discharge	61(57%)	24 (64.8%)	37(52.8%)	0.794 *	30 (56.6%)	31 (57.4%)	0.898 *

RBC: red blood cell; HGB: hemoglobin; HCT: hematocrit; * Chi-Square Test; ** *t* test.

**Table 4:** Blood parameter comparison between age-specific subgroups of CM and AA.

Age Groups	Subgroup	N	RBC (× 106/mm^3^)	HGB (g/dL)	HCT (%)	Anemia at Discharge
0–12 months	CM	16	2.86 ± 0.41	8.67 ± 1.02	25.94 ± 3.87	58%
	AA	18	2.81 ± 0.38	7.78 ± 1.09	23.87 ± 2.92	63%
12–24 months	CM	14	2.93 ± 0.45	8.74 ± 0.98	26.19 ± 4.01	54%
	AA	15	2.88 ± 0.42	7.95 ± 1.12	24.05 ± 3.08	59%
>24 months	CM	23	2.92 ± 0.44	8.50 ± 1.05	25.76 ± 3.98	57%
	AA	21	2.85 ± 0.39	8.02 ± 1.15	24.22 ± 2.85	60%

**Table 5:** Pearson correlation analysis.

Variables	All *n* = 107		Acute Abdomen *n* = 54		Congenital Malformation n = 53	
	Pearson Correlation	*p*-Value	Pearson Correlation	*p*- Value	Pearson Correlation	*p*-Value
HGB baseline × age (months)	−0.469	<0.001	−0.378	0.005	−0.611	<0.001
HGB baseline × HBG after transfusion	0.392	<0.001	0.226	0.052	0.423	0.002

**Table 6:** Multivariate analysis of factors affecting hemoglobin correction post-transfusion.

Variable	Coefficient	Standard Error	*p*-Value
Group (Reference: CM)		12	
Acute Abdomen (AA)	−0.87	0.28	0.004
Age (Months)	−0.02	0.01	0.037
Gender (Reference: Male)			
Female	0.54	0.33	0.112
Baseline Hemoglobin (g/dL)	0.49	0.09	<0.001
Hospitalization Days	0.07	0.03	0.028

**Table 7:** Subgroup analysis of hemoglobin correction by specific condition.

Diagnosis Category	Mean Hemoglobin Increase (g/dL)	*p*-Value
Congenital Malformations		
Gastrointestinal Tract Malformation	3.24	0.052
Central Nervous System Defect	3.67	0.038
Diaphragmatic Hernia	2.98	0.112
Acute Abdomen		
Appendicitis	1.76	0.101
Intestinal Obstruction	1.89	0.093
Peritonitis	2.03	0.078

## Data Availability

The data will be available on request from the corresponding author. The data are not publicly available due to privacy.
